# The Treatment of Wounds

**Published:** 1894-07-07

**Authors:** A. Hanbury Frere


### THE TREATMENT OF WOUNDS.

By A. Hanbury Frere, M.B.

Amid the many advances in the art of surgery in recent years, the management of wounds finds a prominent place. What were at one time scientific assumptions advanced by the few, are now simple truths accepted by the many. One improvement in this direction is the simplification of procedure. There has undoubtedly arisen an endeavour on the part of many to find a short cut to perfection. But in attaining a simplicity of method, there must be no loss of efficiency in the means adopted. "A faultless asepsis," says Gerster[1], and herein lies the gist of the whole matter, "a faultless asepsis has often enabled us to do away both with irrigation and drainage."

Once it was acknowledged that something more was required, in addition to the soap and the scrubbing brush, there arose a whole army of germicides, each of which has in turn, through the vicissitudes of fashion, given way to some more lauded antiseptic, until now there seems a tendency to return to our old friend carbolic acid.

Perhaps the most extensively employed antiseptic at the present time is the bichloride of mercury, or corrosive sublimate solution. Now, great as the value of corrosive sublimate undoubtedly is, in comparison with other antiseptics in general use, yet there are very serious drawbacks attending its employment, which render this agent by no means an ideal antiseptic. Thus Lister[2] tells us that "it cannot penetrate in the slightest degree into anything greasy."

Hence elaborate preparations are necessary in order to cleanse the skin before the bichloride can be employed. This fact detracts somewhat from the value of the otherwise excellent method described by Mr. Higgins.[3]

Again, it has now been proved that the bichloride of mercury, unless used with the greatest care, is dangerous as an intra-uterine injection, from the fact that it is extremely apt to give rise to poisonous symptoms. This tendency on the part of the bichloride to cause mercurialism is no doubt owing to its power to precipitate albumen. Corrosive sublimate when introduced into the system forms with the albumen and sodium carbonate of the blood an insoluble or partially insoluble compound,[4] which may become stored up, and then suddenly absorbed, giving rise to toxic effects. From these facts it is clear that its sphere of usefulness is somewhat limited.

I desire, therefore, to draw attention to the value of a most reliable antiseptic, which is entirely free from the disadvantages attached to corrosive sublimate, and which, in the battle of the bactericides, has not received the attention it deserves. In fact, there seems to be, on the part of many, a failure to appreciate its true worth. Thus, notwithstanding the fact that it is the most powerful germicide known at the present time, it has, until quite recently, been more or less disregarded, mainly, I believe, from a misunderstanding as to its chief properties. I refer, of course, to the biniodide of mercury dissolved in iodide of sodium, so ably advocated by Illingworth, and now pretty largely employed in London under the name of "*Iodic Hydrarg.*"

As I have said, the biniodide of mercury is entirely free from the disadvantages attending the use of the bichloride. Yet this is a fact which is not generally known. Only recently there was in the *British Medical Journal* a discussion on this point, in which Dr. Boxall[5] expressed the opinion that the biniodide of mercury "entails the possible risk of iodism in addition to that of mercurialism." It is difficult to see how Dr. Boxall arrives at this conclusion, but this illustrates very well the kind of misunderstanding that exists in regard to the biniodide of mercury. In the first place, if two powerful agents are introduced into the system in combination, it is not certain that both will give rise to toxic symptoms. Secondly, in the case of the biniodide of mercury in iodide of sodium, such a thing is impossible, since each is soluble in excess of the other. Thus the soluble biniodide of mercury does not, like the bichloride, form an insoluble albuminate; the biniodide, unlike the bichloride of mercury, is very rapidly eliminated, and is not likely to give rise either to iodism or mercurialism. But should any, in view of these facts, still doubt the safety of the biniodide, I may remind them that the best safeguard against mercurialism is the daily administration of small doses of iodide of potassium, as is done in some electrical works with perfect success[6].

My chief reasons for thus calling attention to the biniodide of mercury in iodide of sodium as a lotion for wounds are as follows:—

1. It is easily prepared by the addition of a solution of iodide of potassium or sodium to the liq. hydrarg. perchlor. B.P., until the precipitate which first forms is dissolved. This gives a solution (1 in 1,000) of biniodide of mercury in iodide of potassium or sodium.

2. Having used it extensively in all wounds for several years, I find the results are superior to any obtained with other antiseptics.

3. Its employment on the lines laid down by Dr. Illingworth[7] would considerably diminish hospital expenditure. Its value is especially seen in scalp wounds, in which, under its influence, I have never known suppuration to occur.

4. There can be no doubt that it saves an immense amount of time, trouble, expense, and anxiety to the general practitioner. Having washed the wound with the solution (1 in 1,000), it is sufficient to keep lint applied to the part constantly moist with the same lotion of the strength of 1 in 2,000.

[1] Transactions of American Surg. Assoc., Vol. IX, 1891. [2] B.M.J., Jan. 28, 1893. [3] B.M.J., Ap. 2, 1892. [4] Lancet, Dec. 20, 1890. [5] B.M.J., Nov. 25, 1893. [6] B.M.J., June 11, 1892. [7] Provincial Med. Journ., July, 1890.

